# *α*-Hydroxyketone Synthesis and Sensing by *Legionella* and *Vibrio*

**DOI:** 10.3390/s120302899

**Published:** 2012-03-02

**Authors:** André Tiaden, Hubert Hilbi

**Affiliations:** 1 Competence Center for Applied Biotechnology and Molecular Medicine, University Zürich, Winterthurerstrasse 190, 8057 Zürich, Switzerland; E-Mail: nicki.tiaden@cabmm.uzh.ch; 2 Max von Pettenkofer Institute, Ludwig-Maximilians University Munich, Pettenkoferstrasse 9a, 80336 Munich, Germany

**Keywords:** autoinducer synthase, cell-cell signaling, horizontal gene transfer, pathogen-host interactions, response regulator, quorum sensing, sensor kinase, two-component system, virulence

## Abstract

Bacteria synthesize and sense low molecular weight signaling molecules, termed autoinducers, to measure their population density and community complexity. One class of autoinducers, the α-hydroxyketones (AHKs), is produced and detected by the water-borne opportunistic pathogens *Legionella pneumophila* and *Vibrio cholerae*, which cause Legionnaires’ disease and cholera, respectively. The “*Legionella* quorum sensing” (*lqs*) or “cholera quorum sensing” (*cqs*) genes encode enzymes that produce and sense the AHK molecules “*Legionella* autoinducer-1” (LAI-1; 3-hydroxypentadecane-4-one) or cholera autoinducer-1 (CAI-1; 3-hydroxytridecane-4-one). AHK signaling regulates the virulence of *L. pneumophila* and *V. cholerae*, pathogen-host cell interactions, formation of biofilms or extracellular filaments, expression of a genomic “fitness island” and competence. Here, we outline the processes, wherein AHK signaling plays a role, and review recent insights into the function of proteins encoded by the *lqs* and *cqs* gene clusters. To this end, we will focus on the autoinducer synthases catalysing the biosynthesis of AHKs, on the cognate trans-membrane sensor kinases detecting the signals, and on components of the down-stream phosphorelay cascade that promote the transmission and integration of signaling events regulating gene expression.

## 1. Introduction

Quorum sensing (QS) is a mode of cell-cell communication that allows microbial communities in a given niche to gauge their population density and complexity [1]. QS-dependent processes are based on molecular signaling circuits that synthesize, detect and integrate small diffusible molecules termed autoinducers (AIs) [2,3]. AIs are effective only above a certain threshold concentration, which is reached at a specific cell density of a bacterial population, termed the “quorum”. Accordingly, QS allows bacteria to co-ordinately adjust their behaviour to environmental conditions. AI signaling regulates gene expression of various complex bacterial processes, including biofilm formation, virulence, sporulation, bioluminescence and competence [3]. AI molecules can be grouped into distinct classes according to their basic chemical design. Carbohydrates, lipids (mainly fatty acids), quinolones and small peptides are used as chemical signals in microbial intra- and interspecies communication [4,5].

**Figure 1.**
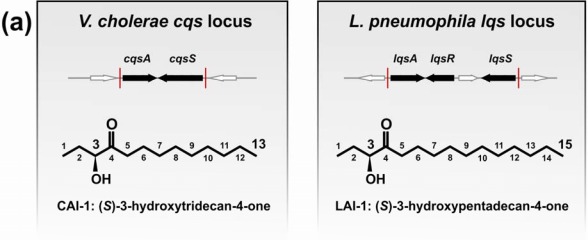

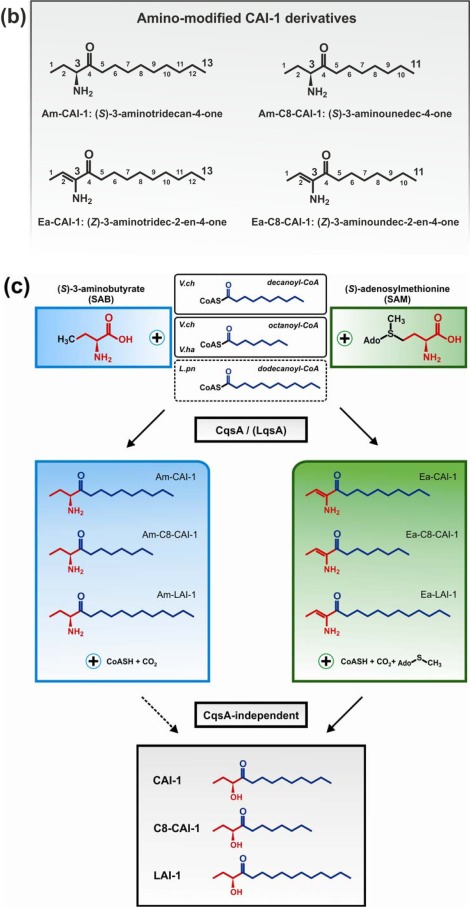
Genetic organization of the *V. cholerae* (*cqs*) and *L. pneumophila* (*lqs*) QS locus, and biosynthesis of AIs produced by CqsA and LqsA. (**a**) The *cqs* and *lqs* loci harbour AI synthases (*cqsA*, *lqsA*), cognate sensor kinases (*cqsS*, *lqsS*) and a response regulator (*lqsR*). AI molecules synthesized by *^V.ch^*CqsA and *^L.pn^*LqsA: (**a**) CAI-1 and LAI-1 AHKs, and (**b**) C3-amino-derivatives of CAI-1 (Am-CAI-1 and Ea-CAI-1). (**c**) Biosynthesis of CAI-1 and C3-amino-derivatives by PLP-dependent *V. cholerae* and *V. harveyi* CqsA and presumably *L. pneumophila* LqsA using (*S*)-3-aminobutyrate (SAB) or (*S*)-adenosylmethionine (SAM) and acyl-CoAs. *^V.ch^*CqsA utilizes decanoyl-CoA (C_10_) or octanoyl-CoA (C_8_) to produce Am-CAI-1/Ea-CAI-1 (C_13_) or Am-C8-CAI-1/Ea-C8-CAI-1 (C_11_). Ea-CAI-1 is converted into CAI-1 by spontaneous hydrolysis and a dehydrogenase. The intermediate Am-CAI-1 is converted into CAI-1 by an unknown mechanism. *^V.ha^*CqsA utilizes only octanoyl-CoA to yield Am-C8-CAI-1/Ea-C8-CAI-1 and C8-CAI-1 (C_11_). Synthesis of LAI-1 (C_15_) by *^L.pn^*LqsA is not elucidated, but might use SAM (or SAB) and dodecanoyl-CoA (C_12_).

Species-specific modifications and derivatives of these basic compounds are found in virtually every bacterial genus, thus yielding a rich microcosm of microbial signal molecules. Many Gram-negative bacteria generate AIs that belong to the first discovered class of *N*-acyl-L-homoserine lactones (AHLs) [2], and/or they produce the almost universally used furanosyl borate diester termed autoinducer-2 (AI-2) [6,7]. Some bacteria utilize additional AIs to regulate gene expression. A class of α,β-unsaturated fatty acids termed diffusible signal factors (DSFs) plays a prominent role in soil- and plant-associated bacteria such as *Xanthomonas*, *Burkholderia* or *Pseudomonas* spp. [8]. Finally, the recently discovered α-hydroxyketones (AHKs) are predominantly produced by aquatic γ-proteobacteria, including *Legionella* and *Vibrio* spp. (Figure 1(a)) [9].

The Gram-negative bacteria *Legionella pneumophila* and *Vibrio cholerae* colonize several environmental niches and are important opportunistic human pathogens that cause the life-threatening diseases “Legionnaires’ pneumonia” or “cholera”, respectively [10,11]. In this review we summarize recent insights into the molecular structure of the *Legionella* and *Vibrio* AHK signaling circuits, discuss the mechanisms of AHK production and sensing and outline their role for pathogen-host interactions, biofilm formation and competence.

## 2. Virulence of *Legionella pneumophila* and *Vibrio cholerae*

*Legionella* and *Vibrio* spp. are water-borne bacteria that persist in aquatic freshwater or brackwater habitats, either as individual planktonic cells, or attached to biotic/abiotic surfaces, where they are part of multispecies biofilm communities [12–14]. Remarkably, *Legionella* and *Vibrio* spp. also evolved specific and distinct mechanisms, which support their survival and growth in eukaryotic hosts ranging from protozoa and nematodes to animals and humans [15–17]. Pathogenic species of both bacterial genera evolved from environmental non-pathogenic progenitor strains by acquiring virulence determinants through horizontal gene transfer [18,19].

### 2.1. Virulence of *Legionella pneumophila*

*Legionella* spp. cause a severe pneumonia called Legionnaires’ disease or a milder, flu-like ailment termed Pontiac fever [20]. The sources for *Legionella* infections can be natural aquatic biotopes, but primarily are anthropogenic water supply systems. Legionellosis occurs through the inhalation of contaminated aerosols, and its epidemiology is strongly linked to the use of technical appliances in developed countries. There are no reports of human to human transmission, and therefore, humans are considered dead-end hosts for *Legionella* species. Approximately half of the more than 50 *Legionella* spp. described so far have been reported to infect humans. Yet, *L. pneumophila* (serogroup 1) and *L. longbeachae* account for the majority of clinical cases [11]. Elderly and immuno-compromised individuals are predominantly prone to Legionnaires’ disease, since their innate immune system is not able to efficiently clear the bacterial infection [21]. In addition to sporadic epidemic outbreaks, nosocomial pneumonia due to *L. pneumophila* is a major issue for public health services around the world [22].

*L. pneumophila* is a facultative intracellular bacterium that thrives within a broad spectrum of environmental phagocytes, including at least 15 protozoan genera, e.g., *Acanthamoeba* or *Hartmanella* spp. and *Dictyostelium discoideum* [16]. The co-evolution of *L. pneumophila* with phagocytic protozoa most likely selected for virulence traits that also allow infection and growth in functionally related professional phagocytes of the human innate immune system, in particular alveolar macrophages [23,24]. The infectious cycle of *L. pneumophila* is a multistage process, where virulent, transmissive bacteria induce their uptake, avoid lysosomal degradation, establish an intracellular replicative niche and finally exit the spent host cell again [25].

A key virulence factor of *L. pneumophila* is a type IV secretion system (T4SS) encoded by 25 different *icm*/*dot* genes. The Icm/Dot T4SS is essential to establish an intracellular replication-permissive niche, termed the *Legionella*-containing vacuole (LCV) [26,27]. A set of more than 250 distinct “effector proteins” is translocated by the T4SS into host cells, where they manipulate conserved cellular processes, such as signal transduction and vesicle trafficking pathways [28–33]. To this end, Icm/Dot-translocated effector proteins target host small GTPases and phosphoinositide lipids, as well as ubiquitinylation and apoptosis factors. Strikingly, many of these effector proteins share sequence homologies with eukaryotic proteins or harbour functional eukaryotic domains, which likely were acquired by trans-kingdom horizontal gene transfer [14,24,34].

### 2.2. Virulence of *Vibrio cholerae*

In contrast to the facultative intracellular bacterium *L. pneumophila*, *V. cholerae* is an extracellular bacterium. The toxigenic *V. cholerae* serogroups O1 and O139 cause the vast majority of cholera cases [35]. Cholera is a dehydrating severe diarrhea, frequently fatal when left untreated. Infection with *V. cholerae* occurs via the consumption of contaminated water or food and subsequent bacterial colonization of the epithelium in the upper small intestine [10]. Poor sanitary standards and uncontrolled dissemination of bacteria by diarrhea into ground and surface water can lead to epidemic outbreaks. *V. cholerae* virulence is a multifactorial process that relies on the interplay of environmentally selected traits such as motility, surface interaction or biofilm formation, combined with the acquisition of phage-derived virulence genes. *V. cholerae* utilizes flagellar motility and secreted proteinases to reach and penetrate the glycokalyx of the intestinal mucosa and binds via N-acetylglucosamine-binding protein A (GbpA) to the epithelium of the small intestine [10]. Efficient colonization of the intestine requires the subsequent expression of the ToxR regulon, which includes the genes coding for two critical virulence factors, the phage-acquired cholera enterotoxin (CTX) and the toxin co-regulated pilus (TCP) [36].

CTX is a secreted AB_5_ subunit protein, which enters intestinal epithelial cells via receptor-mediated endocytosis and disturbs intracellular cAMP signaling. Constitutive cAMP production results in a drastic ion efflux that leads to solute imbalance and massive secretion of water into the intestinal lumen. CTX function is responsible for a profuse secretory diarrhea and is assumed to facilitate transmission of hyper-virulent bacteria [10]. TCP is an auto-interacting type IV pilus that connects *V. cholerae* cells, a process resulting in aggregation and microcolony forming in the mucosa [37]. Importantly, TCP also acts as a receptor for the bacteriophage CTXphi that carries and transduces the CTX genes [38].

Once established, *V. cholerae* proliferates in the nutrition-rich small intestine. At late stages of the infection, parameters such as host-specific stimuli, availability of nutrients and increasing bacterial cell density coordinate an “exit response” that promotes detachment of the bacteria, survival in the aquatic environment and spread to new hosts [10]. This stage involves QS-dependent repression of ToxR-controlled genes, activation of the haemagglutinin/protease HapA and the motility apparatus [39,40].

## 3. QS in Vibrio and *Legionella* spp

The facultative pathogenic potential of *V. cholerae* and *L. pneumophila* implies a biphasic life style and a periodic transition between environmental and host-associated niches [10,14]. The transition between different habitats is reflected by a prominent switch in gene expression that leads to niche-specific expression patterns [24,41]. Accordingly, *V. cholerae* and *L. pneumophila* reciprocally express genes, which either support bacterial growth under distinct environmental conditions, or promote host colonization and virulence. Under laboratory conditions, these transcriptional programs can be (at least partially) mimicked in broth cultures. In *V. cholerae* the reciprocal gene expression pattern is responsive to the cell density and, via cAMP and cAMP receptor protein (CRP), also dependent on the metabolic state [3,42,43]. In *L. pneumophila* the biphasic gene expression is regulated by the growth phase [24,25]. Noteworthy, in both species the transition between niche-specific gene expression patterns is dependent on two-component (TC) systems (VarA/VarS in *V. cholerae*, LetA/LetS in *L. pneumophila*) and the global regulator CsrA [44–48]. The VarAS-CsrA or LetAS-CsrA systems integrate metabolic signals such as nutrient depletion and carbon availability, which are strongly linked to the bacterial cell density and growth phase.

Colonizing a new niche provides the opportunity to exploit different nutrition-rich sites, yet the bacteria also have to continuously adapt to changing environmental conditions. Accordingly, *V. cholerae* and *L. pneumophila* need to integrate complex patterns of environmental and host-derived stimuli to ensure efficient replication within a niche, progression to subsequent infection stages within a host and transition between ecological niches. To this end, a fine-tuned sensor machinery is essential to constantly monitor endogenous and environmental cues, transmit and integrate the signals and respond by regulating gene expression. *V. cholerae* and *L. pneumophila* employ QS circuits in concert with additional sensor systems to coordinate various processes during their life cycle, including pathogen-host interaction and production of virulence factors [39,49–51], biofilm and filament formation [40,52–54], the regulation of a genomic “fitness island” [54] and natural competence [55–57].

The molecular structure of QS signaling units basically comprises an AI synthase and a cognate sensor protein, which is coupled to a signal transduction phosphorelay that links the detection of AIs to gene regulation [3]. QS circuit components differ significantly between distinct systems and bacterial species. LuxI/LuxR-like systems, first described in *Vibrio fischeri*, synthesize and detect freely diffusible AHLs [58,59]. Here, the AHL ligand binds the LuxR regulator directly in the cytosol and, depending on the system, either destabilizes or stabilizes LuxR dimerization and DNA binding [2]. Other QS systems adopt membrane-bound sensor kinases derived from TC systems to detect and transmit an AI signal, e.g., the *Vibrio*-specific LuxM/LuxN AI synthase/sensor kinase system that produces and responds to an AHL compound termed harveyi autoinducer-1 (HAI-1), or the broadly-used LuxS/LuxPQ system that signals through AI-2 [3].

Bacterial TC systems are sensor units composed of an inner membrane-associated sensor histidine kinase and a downstream response regulator [60]. TC sensor proteins recognize a wide range of chemical and physical stimuli in the periplasmic space, including osmolarity, pH, envelope damage, as well as chemical signaling molecules. The common feature of these bacterial sensors is the conversion of ligand binding into phosphorylation events, which are integrated and transduced via phosphorelay pathways to downstream regulatory proteins [61,62]. The genomic organization of TC genes, encoding a sensor kinase and a response regulator, can either constitute tandem pairs controlled as an operon or represent distantly located “orphans” used by different transcriptional programs [63,64]. QS genes, encoding a AI synthase and a cognate sensor protein, are usually located adjacently or in close vicinity of each other in the bacterial genome, and therefore, are believed to retain their pairwise functional relationships through co-evolution as a single cassette [65,66]. Multiple sensors of TC and QS systems can be integrated and may converge on shared response regulators.

## 4. AHK Signaling in *V. cholerae* and *L. pneumophila*

### 4.1. Components and Integration of AHK Signaling

While AHL and AI-2 signaling circuits are broadly used by many bacterial species [67,68], the usage of AHKs as small signaling molecules is apparently restricted to a group of aquatic γ-proteobacteria, including *Vibrio* and *Legionella* spp. [9]. The *V. cholerae* and *L. pneumophila* AHK signaling circuits are constituted by the *cqs* or *lqs* (*c**holera/**L**egionella* quorum sensing) gene clusters, respectively [9]. These clusters encode cognate pairs of an AI synthase and a sensor histidine kinase (CqsA/CqsS, LqsA/LqsS), which produce and detect the corresponding AHKs CAI-1 (*c**holera* autoinducer-1) [69] or LAI-1 (*L**egionella* autoinducer-1) [70] (Figure 1(a)). In addition, the *lqs* gene cluster encodes the putative response regulator LqsR [50].

*Vibrio* spp. utilize the Cqs system together with additional QS systems. *V. cholerae* and *V. harveyi* use two or even three QS systems operating in parallel to integrate AI signals in a joint phosphorylation cascade. Thus, *V. cholerae* employs CqsA/CqsS, as well as LuxS/LuxPQ to signal through CAI-1 and AI-2 [6,49,68,69]. In addition to these two systems, *V. harveyi* uses a third, LuxM/LuxN-type system, which signals through the AHL molecule HAI-1 [71,72]. QS signals received by all sensor kinases (CqsS, LuxQ and LuxN) are channelled onto the phosphorelay protein LuxU that transfers the phosphate to the response regulator LuxO [73,74].

**Figure 2.**
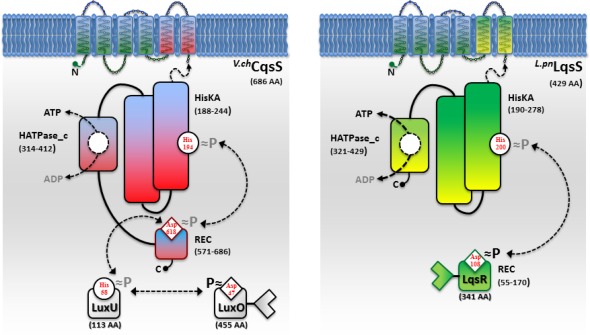
Signal transduction by the *V. cholerae* CqsS and *L. pneumophila* LqsS sensor kinases. *^V.ch^*CqsS and *^L.pn^*LqsS are six trans-membrane helix sensor kinases with C-terminal cytoplasmic signal transduction domains. Predicted sub-domains (amino acids in parenthesis): HisKA (histidine kinase A domain with conserved histidine phospho-acceptor site), HATPase_c (catalytic ATP binding and transferase domain, C-terminal); REC (receiver domain with conserved aspartate). CqsS is a hybrid histidine kinase coupled to a phosphorelay system. The HisKA/HATPase_C domain catalyzes the autophosphorylation by ATP at H194 and the phosphotransfer to D618 in the REC domain. The phosphoryl group is then shuttled via the orphan phosphorelay protein LuxU (H58) to the REC domain of the response regulator LuxO (D47). In LqsS, after phosphorylation of H200, the phosphate is presumably transferred to D108 in the REC domain of the response regulator LqsR. CqsS and LqsS are likely bifunctional kinases/phosphatases.

A common feature of the sensor histidine kinases CqsS, LuxQ and LuxN is their ability to switch between kinase and phosphatase function. The LuxQ [75] and LuxN [76] sensor kinases have been instrumental to study mechanistic aspects of coupling the binding of an AI ligand to the switch from kinase to phosphatase activity. High-resolution crystal structures of the periplasmic binding protein LuxP and a fragment of the sensor kinase LuxQ in complex with or without its ligand AI-2 revealed that binding of AI-2 to LuxP disrupts tetrameric complexes of LuxPQ dimers, thus causing a switch from kinase to phosphatase activity. Similarly, a switch from kinase to phosphatase activity is also proposed for CqsS upon interaction with its ligand CAI-1 (Figure 2). However, the exact mechanism of how CAI-1 binding alters the oligomerization state and the activity of a putative CqsS dimer has to be elucidated. At low AI concentration the sensor kinase activity will lead to phosphorylation and activation of the downstream targets LuxU and LuxO. Conversely, at high AI concentration the sensor phosphorylation activity will result in the dephosphorylation and inactivation of LuxU and LuxO.

Below the AI threshold concentration, phosphorylated LuxO represses the activity of the QS master regulator HapR in *V. cholerae* [49] or LuxR in *V. harveyi* [77], respectively. The repression of HapR is achieved via coordinated LuxO-RpoN-Fis-dependent expression of four functionally redundant small regulatory RNAs termed Qrr1-4. The Qrr sRNAs act in concert with the RNA chaperonine Hfq to destabilize *hapR* mRNA and prevent its translation [45,78–80]. In contrast, high AI concentrations deactivate LuxO and release the repression of HapR and LuxR. At high AI concentration, virulence and biofilm formation in *Vibrio* spp. is repressed by HapR and LuxR, while the haemagglutinin/protease HapA, motility and competence in *V. cholerae* or bioluminescence in *V. harveyi* are induced [3,10,55].

The genes for LuxU, LuxO and HapR are not associated with the *cqs* genes or the other QS genes in the *V. cholerae* genome. This scattered genomic organization reflects the shared usage of the genes by parallel QS systems in *Vibrio* spp. Thus, LuxU, LuxO and HapR are also targets for other sensor or regulatory system systems, including the VarAS-CsrA system, VpsRS and cyclic-di-GMP, which converge on LuxO and control HapR-regulated genes [45,81,82].

In contrast to *Vibrio* spp, *L. pneumophila* apparently employs only the AHK-based Lqs QS system. The *lqs* genes are not present in the *L. longbeachae* genome [83], and *lqsA* was not detected by PCR using genomic DNA of several non-pneumophila strains [70]. In *L. pneumophila* AHK signaling is assumed to be mediated, at least partially, by the putative response regulator LqsR [50] (Figure 2). This prototypic member of a novel family of response regulators harbours a canonical, N-terminal receiver domain (amino acids 80–160) including the conserved aspartate residue (D108). The C-terminal fragment of LqsR does not show homology to any known signal output domains, and its function is unknown. The clustering of the *lqsR* gene with *lqsA* and *lqsS* is conserved in bacterial species that harbour an LqsR homologue, suggesting an evolutionarily conserved functional correlation between LqsA/LqsS and LqsR [9,50]. Similar to the modification of *Vibrio* LuxO function by a range of regulatory factors, LqsR is also intimately connected to other regulatory networks. The production of LqsR is dependent on the stationary sigma factor RpoS, which in the *L. pneumophila* life cycle controls the reversible transition from the replicative to the virulent phase, as well as pathogen-host interactions [9,50].

*L. pneumophila* strains lacking *lqsS* or *lqsR* show severe defects for uptake by phagocytes, intracellular growth and the production of extracellular filaments [50,54]. In contrast, *L. pneumophila* lacking *lqsA* is only mildly impaired for host cell infection, similar to *V. cholerae* lacking *cqsA*. Yet, the overexpression of *lqsA* in *L. pneumophila lqsS* or *lqsR* mutant strains restores the virulence defects of these mutant strains to wild-type levels. These results suggest that the LqsA product LAI-1 indeed regulates virulence and that *L. pneumophila* employs multiple and redundant LAI-1-responsive signaling pathways [54]. It is tempting to speculate that the LCV represents a LAI-1-impermeable compartment and that the cell density-dependent concentration of AHK signaling molecules within this pathogen vacuole regulates bacterial virulence and motility, as well as cycling from the transmissive to the replicative growth phase.

### 4.2. CAI-1/LAI-1 AHK Signal Production by CqsA/LqsA AI Synthases

The CqsA/CqsS circuit signals through the major *V. cholerae* AI, the AHK molecule CAI-1 (3-hydroxytridecan-4-one) [69], whereas the *L. pneumophila* LqsA/LqsS system mainly produces and presumably responds to LAI-1 (3-hydroxypentadecan-4-one) (Figure 1(a)) [70]. The AI synthases CqsA and LqsA are 45% identical, and both enzymes are predicted to be related to pyridoxal-5′-phosphate (PLP)-dependent aminotransferases [49,50,69]. Accordingly, CqsA and LqsA contain conserved lysine residues (CqsA_K236, LqsA_K258), which covalently bind PLP [70,84,85]. Moreover, LqsA and CqsA are also functionally similar, as *lqsA* partially complements the deletion of *cqsA* in *V. cholerae* [70].

Crystallographic and biochemical characterization of CqsA confirmed its function as a PLP-dependent aminotransferase-like enzyme [84,85]. Studies with purified CqsA revealed that *in vitro* the enzyme catalyses the formation of a carbon-carbon bond between (*S*)-2-aminobutyrate (SAB) and decanoyl-Coenzyme A (CoA) to produce (*S*)-3-aminotridecan-4-one (Am-CAI-1) (Figure 1(b,c)). Synthetic Am-CAI-1 and CAI-1 both bind to CqsS, as detected by a bioluminescence assay. However, CAI-1 is the prominent AHK compound in *V. cholerae* culture supernatants, suggesting that Am-CAI-1 is an intermediate, which is converted to CAI-1 by the bacteria [84].

In recent studies dissecting the biosynthetic route of CAI-1, (*S*)-adenosylmethionine (SAM) was identified as a much more efficient biosynthetic co-substrate than SAB for CqsA to produce the novel compound 3-aminotridec-2-en-4-one (Ea-CAI-1) (Figure 1(b,c)) [86,87]. Isotope labelling experiments confirmed that Ea-CAI-1 is synthesized by CqsA *in vitro*, as well as *in vivo* by coupling SAM and decanoyl-CoA. The unstable enamine Ea-CAI-1 is converted to CAI-1 via the spontaneous conversion into tridecane-3,4-dione (DK-CAI-1) followed by an NADPH-dependent reduction catalysed by a dehydrogenase [86,87]. The use of SAM as a co-substrate in the synthesis of AHKs is interesting, as it indicates that bacteria employ this abundant metabolite for the synthesis of at least three classes of QS signaling molecules: AHKs, AHLs and AI-2. The abundance and physiological importance of SAM poses several advantages for its usage as an AI substrate, since (i) this metabolite represents a constant source of QS signals and thus is a reliable parameter for cell density, and (ii) it reflects the physiological status of a bacterial population due to its prominent role in housekeeping reactions [86]. Thus, SAM-based QS systems link an important parameter of bacterial physiology to population density.

In addition to CAI-1 derivatives harbouring a C_10_ acyl tail (Am-CAI-1, Ea-CAI-1, DK-CAI-1, CAI-1), *V. cholerae* produces and responds to AHKs with other acyl tail lengths [69,70,84]. *V. cholerae* CqsA shows promiscuous substrate specificity and also forms Ea-C8-CAI-1 from SAM and octanoyl-CoA. Still, *V. cholerae* CqsA prefers the acyl CoA substrate C_10_-CoA over C_8_-CoA, and this substrate preference is also reflected by the *in vivo* production profiles of CAI-1 molecules detected in native cultures supernatants (concentration ratios of C8-CAI-1:Ea-CAI-1:CAI-1 = 1:7.4:11.8).

Relaxed specificity is also observed for *L. pneumophila* LqsA, which produces either LAI-1 (C_12_ acyl tail), or to a smaller extent CAI-1 (C_10_ acyl tail) and derivatives with C_11_ or C_13_ acyl tails [70]. However, this relaxed product specificity was observed upon heterologous production of LqsA in *E. coli*. The physiological specificity of LqsA might be more stringent, and presumably, dodecanoyl-CoA is used preferentially by *L. pneumophila* as a co-substrate to synthesize LAI-1(Figure 1(c)). The *in vivo* pathway for the biosynthesis of LAI-1 in *L. pneumophila* has yet to be elucidated, and it is not known, whether LqsA also produces Ea-LAI-1 or Am-LAI-1.

In contrast to the relaxed specificities of *V. cholerae* CqsA and *L. pneumophila* LqsA for different CoA co-substrates, the *V. harveyi* CqsA homologue exhibits a rather stringent substrate specificity, using only octanoyl-CoA together with SAM or SAB to produce Ea-C8-CAI-1 and C8-CAI-1, respectively [87]. In line with the stringent AI production, *V. harveyi* CqsS preferentially detects C8-CAI-1 derivatives.

### 4.3. AHK Signal Perception by CqsS/LqsS Sensor Kinases

**Figure 3.**
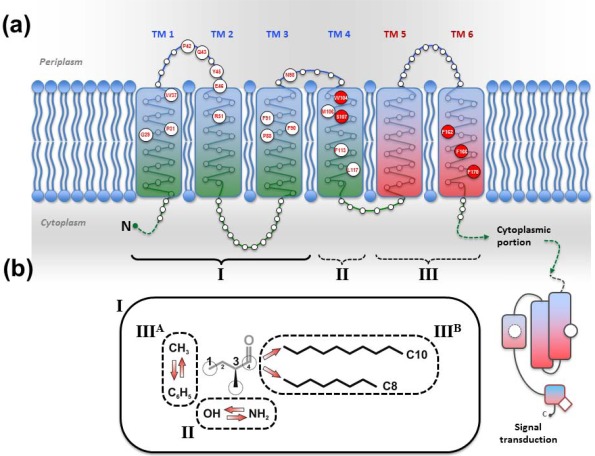

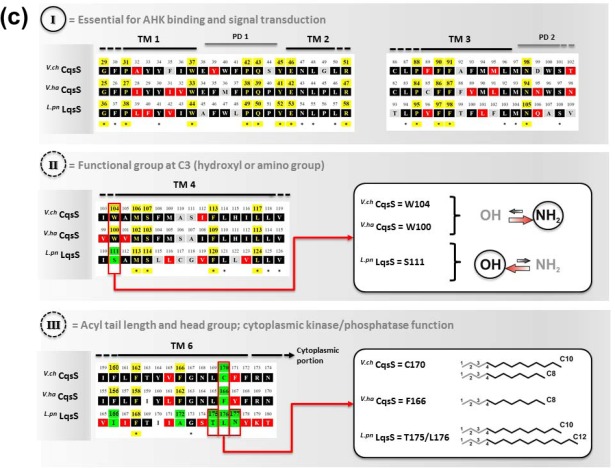
Signal recognition by the *V. cholerae* CqsS and *L. pneumophila* LqsS sensor kinases. (**a**) Topology model of the *V. cholerae* CqsS trans-membrane sensor domain based on the secondary structure prediction algorithms TMPRED and TMHMM. The N-terminal sensor domain encompasses six trans-membrane α-helices and defines the specificity and sensitivity for distinct AHK molecules (enlarged circles, amino acid position and one-letter code). (**b**) Sensor domain regions (I, II, III) define the interactions with functional groups of AHK ligands. (I) TM 1–3 and periplasmic domain PD 1 contain conserved amino acid clusters essential for ligand binding and signal transduction (cytoplasmic domain CD 1). (II) TM 4 (W104, S107) discriminates between hydroxyl and amino groups at C3 of AHK ligands. (III) TM 6 (F162, C170) determines binding to the head group (methyl *vs*. phenyl) and the acyl tail of AHK ligands. (**c**) Amino acid sequence alignment (Clustal Omega algortithm) of the sensor domains of *V. cholerae* (*V.ch*) CqsS, *V. harveyi* (*V.ha*) CqsS and *L. pneumophila* (*L.pn*) LqsS. The positions of species-specific amino acids in the sensor domain regions (I, II, III) are indicated, and stars denote conserved amino acids. Color scheme: black (2/3 conserved); red (similar amino acids); grey (different amino acids); yellow (amino acids defining ligand sensitivity, specificity and signal transduction motifs); green (amino acid exchange results in altered ligand specificity). Boxes illustrate altered ligand preferences due to site-specific amino acid polymorphisms in sensor domain region II and III.

The CAI-1 receptor CqsS and the potential LAI-1 receptor LqsS belong to the class of six trans-membrane helix TC sensor histidine kinases (Figure 3(a)). CqsS and LqsS couple the detection of the AI molecules via a receptor domain at the N-terminus to signal transduction modules at the C-terminal part of the protein [9]. TC sensor proteins are mainly located in the inner bacterial membrane, where they sense specific environmental signals present in the periplasmic space [62]. However, the molecular nature and the exact mechanisms of how these signals interact with the corresponding TC sensor kinases are poorly understood. Only a small fraction of TC system ligands has been identified so far, and therefore, AI molecules such as AHKs are valuable research tools to study membrane-bound sensors [3].

Structure-function and ligand interaction analysis of intrinsic polytopic membrane proteins is challenging, yet recent studies used an elegant biochemical/genetic strategy to investigate CAI-1 recognition by CqsS sensor kinases [87–89]. The pairwise approach is based on defined CqsS sensor mutants harboring amino acid substitutions at distinct positions within the six trans-membrane helices, combined with probing these mutant receptors with natural and modified CAI-1 molecules. This approach allowed the identification of sensitivity and specificity determinants of CqsS that govern the discrimination between distinct CAI-1 molecules. The first three trans-membrane helices were found to harbor motifs essential for ligand binding and signal transduction. These motifs are conserved in all *Vibrio* CqsS homologues and in *L. pneumophila* LqsS, suggesting that they constitute a common ligand-binding domain (Figure 3(a)).

Some motifs in the first half of the N-terminal receptor region are strictly conserved, indicating an interaction with chemical characteristics present in all AHK molecules, such as the C3/C4 modifications in the hydrophilic head group and the hydrophobic acyl tail. The second half of the CqsS receptor domain is more variable among different *Vibrio* spp. and thus defines the binding specificity of the receptor for distinct natural and synthetic CAI-1 analogues. The amino acids W104 and S107 in *V. cholerae* CqsS determine the specificity for the CAI-1 head group, such that the amino modification at position C3 is (slightly) favored over the hydroxyl modification (Figure 3(b)). By contrast, any alteration of W104 results in an increased preference for a hydroxyl over an amino modification at the C3 position [88]. Moreover, the residues F160/F162/F166 and C170 in *V. cholerae* CqsS determine the size of the polar head group and the length of the acyl tail of the AHK ligand to be bound. This proposed “gatekeeper” function likely controls the access to the binding site, thereby preventing cross-talk between related signals and reducing signal to noise from similar molecules in the environment. A gatekeeper is an essential regulatory mechanism for sensing systems, because it prevents inappropriate activation or inhibition of signaling, and thus, maintains the stringency of QS input and responses [3]. The motif F160/F162/F166 is conserved in *Vibrio* spp., and F162 is essential for proper interaction with the head group of CAI-1 molecules. Pairwise screening of CqsS sensor mutants with a synthetic library of modified CAI-1 analogues showed that amino acid exchanges at residues F160/F162/F166 can facilitate the binding of inhibitory CAI-1-like molecules carrying a phenyl moiety at the head group. These mutants are impaired for downstream kinase activity, and thus, residues F160/F162/F166 are suggested to act in concert to prevent binding of inhibitory non-natural CAI-1 molecules [88].

The position corresponding to *V. cholerae* C170 is highly variant in different *Vibrio* spp. *V. cholerae* C170 determines the preference for a C_10_ acyl tail, but with lower affinity also accepts a C_8_ acyl tail (Figure 3(b)). Thus, in line with the promiscuous substrate selection of *V. cholerae* CqsA utilizing C_10_-CoA as well as C_8_-CoA [86], *V. cholerae* CqsS displays a relaxed ligand specificity. Yet, *V. cholerae* CqsS does not recognize LAI-1 with a C_12_ acyl tail [70]. Conversely, the strict substrate specificity of *V. harveyi* CqsA is reflected in the presence of a bulky phenylalanine at the corresponding position (F166), which only allows binding of CAI-1 derivatives with a C_8_ but not with longer acyl tails [87]. Furthermore, *V. harveyi* CqsS preferentially binds Ea-C8-CAI-1, and the presence of the enamine group at the C3 moiety is critical for downstream signaling.

Direct binding and stimulation of the *L. pneumophila* sensor kinase LqsS by LAI-1 has not been shown experimentally. However, in analogy to *V. cholerae* CqsS, the motifs located in the first three trans-membrane domains that are essential for the interaction with CAI-1, are conserved at the corresponding sites in LqsS (Figure 3(a)). Moreover, the motifs in the second part of the CqsS sensor domain, which define the chemical characteristics of bound AHKs, are also similar to the corresponding motifs in LqsS. Accordingly, the serine at position 107 is conserved in the sensor kinases in *Vibrio* spp., as well as in *L. pneumophila* (S114). In contrast, the tryptophan at position 104 in CqsS is replaced by a serine in LqsS (S111), which possibly indicates a preference for LAI-1 molecules with hydroxyl modifications. Furthermore, at the position that discriminates acyl tail length in *Vibrio* spp. (C170 in *V. cholerae* CqsS, F175 in *V. harveyi* CqsS), LqsS harbors small amino acids (T175/L176), suggesting that the LqsA/LqsS system might show relaxed specificity similar to *V. cholerae* CqsA/CqsS. Finally, LqsS and CqsS also differ in the gatekeeper motif, which defines binding of the polar head group (F160/F162/F166 *vs*. I166/F168/A172).

In summary, the *V. cholerae* CqsA/CqsS system produces and detects three different AHK molecules: (1) CAI-1, (2) amino-modified CAI-1 analogues (Am-CAI-1, Ea-CAI-1), and (3) Ea-C8-CAI-1 (but not C8-CAI-1). *V. cholerae* CqsA prefers the acyl CoA substrate C_10_-CoA over C_8_-CoA, and this substrate preference also correlates with the ligand selectivity of CqsS, which binds CAI-1 with C_10_ acyl tails more efficiently than molecules with C_8_ acyl tails. The chain length discrimination is defined by the amino acid residue at position 170 located in the sixth trans-membrane helix of the CqsS receptor domain. *V. cholerae* C170 allows the detection of ligands with C_10_ or C_8_ but not shorter (C_6_, C_4_) or longer (C_12_) acyl tails. The motifs F160/F162/F166 (polar head group) and W104/S107 (functional group at C3 moiety) ensure the exclusive binding of CAI-1 analogues with a relaxed specificity for amino and hydroxyl modification.

## 5. Ecological and Evolutionary Implications of AHK Signaling

The *V. cholerae* CqsA/CqsS system shows a rather relaxed signal discrimination, and multiple CAI analogues can interact with the sensor kinase CqsS, including molecules produced by other *Vibrio* spp. Since *V. cholerae* and *V. harveyi* produce and detect Ea-C8-CAI-1, this CAI-1-derivative might be used for communication among different *Vibrio* spp. Potential ecological interactions among *Vibrio* spp. might indeed take place in river deltas and other aquatic ecosystems [10]. Still, the CqsS sensors of different *Vibrio* spp. are most sensitive for AHK molecules produced by their cognate CqsA AI synthases. The coordination and fine-tuning of cognate AI synthases and AI receptors ensures efficient ligand-receptor interactions, avoids inappropriate communication and maintains high fidelity QS signaling.

*V. harveyi* produces higher concentrations of the hydroxyl-modified C8-CAI-1 compared to Ea-C8-CAI-1, even though the hydroxyl molecule is only a weak agonist. Similar CAI-1 production profiles were found in supernatants of several other marine *Vibrio* spp., e.g., *V. parahaemolyticus*, *V. alginolyticus* or *V. anguillarum* [72,87]. Furthermore, all these *Vibrio* spp. employ three parallel QS systems (*i.e.*, CqsA/CqsS, LuxS/LuxPQ and LuxM/LuxN). As LuxS/LuxPQ-based AI-2 signaling is broadly used, the stringency imposed by the AHK (and AHL) systems might be essential for an accurate integration of multiple and similar AI signals.

In contrast to *Vibrio* spp., which employ several QS systems, *L. pneumophila* apparently uses only the Lqs system. Perhaps, this reflects the distinct lifestyles of the bacteria. While *Legionella* spp. can colonize extracellular niches, such as multi-species biofilms, their preferential habitat is likely the resident amoebal fauna in biofilms [14,90]. Thus, intracellular *L. pneumophila* might avoid competitors, which are normally encountered in nutrition-rich extracellular ecological niches, and consequently, the bacteria might have lost (or never acquired) QS systems that support broad inter-species communication. In agreement with the notion that (inter-species) communication systems are dispensable for intracellular bacteria, neither *L. longbeachae* [83], nor *Legionella* spp. other than *L. pneumophila* apparently possess the *lqs* system [9,70,91].

The *lqs* genes are located in a genomic region of *L. pneumophila* that seems to represent a hot spot for recombination. The corresponding site in *L. longbeachae* harbours a homologue of the putative effector gene *legG2* (llo0327, lpg0267), which is flanked by transposase elements and localizes upstream of a homologue of *E. coli hdeD*. The function of *L. pneumophila hdeD* is unknown [54], but interestingly, the gene interrupts the convergently transcribed *lqsS* and *lqsR* genes (Figure 1(a)) [50]. Thus, the discrete genes for *lqsS* and *lqsR* might originate from a larger *cqsS*-like ancestor gene, which was split by the resident *hdeD* locus by recombinatory events that integrated a *cqs*-like cassette into the *L. pneumophila* genome.

Gene clusters including homologues of *lqsA* and *lqsS* are present not only in *L. pneumophila* and *Vibrio* spp., but also in several other environmental bacteria, including *Ralstonia eutropha*, *Nitrococcus mobilis*, *Burkholderia xenovorans* and *Polaromonas* spp. [9,50]. While most of these gene clusters harbor only an *lqsA* and *lqsS* homologue, some also contain an *lqsR* homologue, but the *hdeD* gene is present only in the *L. pneumophila lqs* cluster. Likely, *L. pneumophila* acquired the *lqsA*-*lqsR*-*lqsS* cluster by horizontal gene transfer from other environmental bacteria, such as *Nitrococcus*, *Burkholderia* or *Polaromonas* spp. The prevalence of putative AHK AI synthases and sensors suggests that intra- and inter-species AHK-based cell-cell communication is wide-spread among bacteria.

## 6. Conclusions/Outlook

The water-borne opportunistic pathogens *L. pneumophila* and *V. cholerae* harbor the *lqs* or *cqs* gene clusters and engage in cell-cell communication through the production and sensing of the AHKs LAI-1 or CAI-1, respectively. These small signaling molecules regulate virulence and pathogen-host interactions, formation of biofilms or extracellular filaments, competence, and the expression of a genomic “fitness island”. Recent studies elucidated the PLP-dependent biosynthesis of CAI-1 and amino-derivatives by the AI synthase CqsA using SAM and decanoyl-CoA as substrates. CAI-1-related AIs and probably also the corresponding LAI-1 molecules signal through CqsS/LqsS sensor kinases via a phosphorelay cascade, which converges on the *V. cholerae* master regulator HapR or the *L. pneumophila* response regulator LqsR. The continued analysis of the Lqs and Cqs QS systems (i) yields insights into a QS strategy likely used by a number of environmental bacteria, (ii) provides tools to analyze the mechanism of signal transduction by AHK ligands and TC system sensor kinases, and (iii) might lead to the development of compounds useful in clinical or environmental settings to interfere with virulence and persistence of important human pathogens.
